# Building and Growing a Hospital Intranet: A Case Study

**DOI:** 10.2196/jmir.3.1.e10

**Published:** 2001-03-17

**Authors:** Kenneth R Ong, Michelle Polkowski, Geoff McLemore, Mark Greaker, Malcolm Murray

**Affiliations:** ^1^Saint Vincent's Catholic Medical CentersDepartment of Information SystemsUSA

**Keywords:** Intranet, Internet, Hospital, Medical Informatics, Healthcare Informatics

## Abstract

**Background:**

The Intranet is a rapidly evolving technology in large hospitals. In this paper, we describe the first phase of an Intranet project in a multi-hospital system in New York City.

**Objectives:**

(1) To encourage the use of the Intranet among physicians, nurses, managers, and other associates in a multi-hospital system; and (2) to build the Intranet in a cost-effective manner using existing resources.

**Methods:**

A WebTrends Log Analyzer assessed the Intranet use in terms of the number of accesses from each department.

**Results:**

A broad range of features, including medical knowledge resources, clinical practice guidelines, directions, patient education, online forms, phone directory, and discussion forums were developed. Analysis of more than 890,000 hits revealed the departments with hits greater than 1,000 were the 'Library' (6,130), 'Physicians Gateway' (2,539), 'Marketing' (1,321), 'Information Systems' (1,241), and 'Nutrition' (1,221). Of 819 unique visitors, 74 per cent visited more than once.

**Conclusions:**

It is possible to create and diffuse an Intranet in a multi-hospital system in a cost-effective manner. However, the key challenges were selling the potential of this new technology to opinion leaders and other stakeholders, and converting pre-existing printed content by obtaining word processed and image files from other departments or contracted print publishers.

## Introduction

Hospitals are rapidly adopting Intranet technology. According to a survey by PriceWaterhouseCoopers and Zinn Enterprises, the proportion of large hospitals (number of acute care beds greater than 500) with an Intranet rose from less than half of the respondents in 1999 to nearly three-quarters in 2000 [1].

This paper describes the first phase of Intranet growth in a hospital system from October 1999 through July 2000. We describe the challenges encountered, solutions created, and resources brought to bear to develop a dynamic, enterprise-wide Intranet.

### Background

Saint Vincent's Catholic Medical Centers of New York (SVCMCNY) is a newly merged enterprise of seven acute care hospitals with services that include a wide spectrum of health care. The system includes 2,600 acute medical/surgical beds, 61 primary care, behavioral health and ambulatory care sites, 800 long-term care beds, 1 million home care visits, approximately 2,000 physicians, and 15,000 associates. SVCMCNY serves communities in Brooklyn, Manhattan, Queens, Staten Island, and Westchester.

The first phase of the Intranet project was limited to the services and settings of care affiliated with four acute care hospitals in the boroughs of Brooklyn and Queens in New York City.

## Methods

### Resources, Infrastructure, and Software

The Intranet comprises a variety of resources, infrastructure, and software (Table 1).

The Intranet project team comprises a project manager, a webmaster, and a technical lead. At present, the webmaster also designs and develops the Intranet. Departments, with sites on the Intranet, have content providers that send digital content to the developer.

Internet Explorer 5 is the standard web browser and FrontPage 2000 the supported HTML (hypertext markup language) editor.

Each department with an Intranet site has a content provider, who must ensure that the relevant department chair has approved material before it is sent to the developer. Content is sent via e-mail. Hardcopy photography is accepted but paper-based text for optical character recognition is not.

Site statistics are monitored with WebTrends Log Analyzer [2] and FrontPage 2000's report function.

### Site Map and Content

At the end of this first phase of the project, the Intranet has 267 MB of files. At the time of writing, 3,011 files have been posted in the last 30 days. The remaining 1,230 files have not been modified in over 72 days. Of the 5,447 hyperlinks, the majority (5,088) is internal and a minority (359) is external.

**Figure 1:**
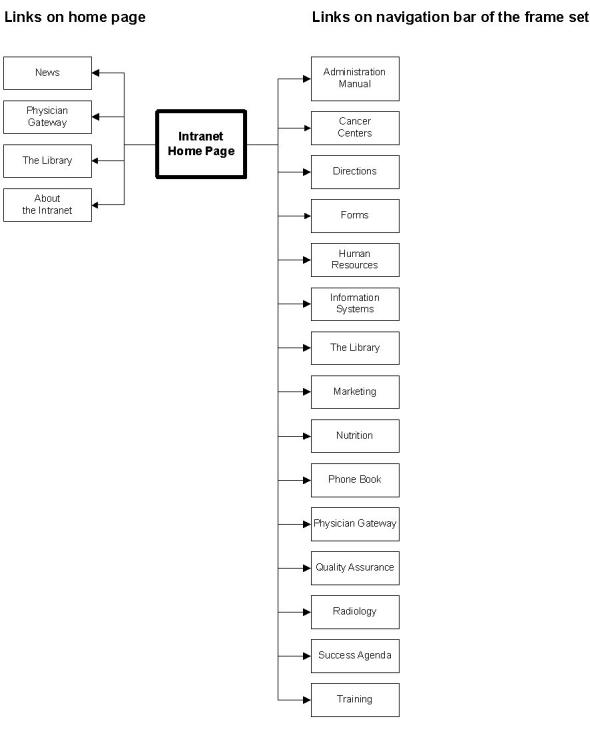
Example Frameset

A frameset is used to facilitate navigation with links to the main department sites, such as the 'Administrative Manual', 'Library', 'Phone Book', and the 'Physician Gateway' (Figure 1).

The home page has links to a 'News' page with the latest corporate announcements. For example, the current version of 'News' has information and links to resources on Ambulatory Payment Classification and a news release about a prostate cancer screening campaign.

Functions served by the Intranet include those shown in Textbox 1.

**Table 1:** Intranet Resources, Infrastructure, and Software

*Category*	Description		
*Resources*	*Position*	*Intranet Project Team Role*	*Time devoted to Intranet (full-time equivalent, FTE)*
	Director, Medical Informatics	Design, development, and webmaster	0.5
	Director, Data Administration and Security	Project manager	0.1
	Network Coordinator	Technical lead	0.1
*Infrastructure*	Compaq Proliant 3000 Server with 256 MB RAM and 14 GB hard disk Compaq Proliant 3000 Server with 256 MB RAM and 14 GB hard disk		
*Software*	Windows NT 4 Microsoft Internet Information Server Internet Explorer 5 FrontPage 2000 Microsoft Office Suite Image Composer Adobe Photo Deluxe, Business Edition WebTrends Log Analyzer Hewlett Packard Precision Scan Pro

**Functions served by the Intranet of the Saint Vincent's Catholic Medical Centers of New York**

- Online administrative manual
- Internal marketing
- Directions to the hospitals
- Digital forms
-
  - Adult Medical Record Review Form
  - Inter-Library Loan Request
- Form repository (links to Microsoft Word files)
- Secure site for an enterprise-wide work group ('Success Agenda')
- Public and restricted information for Human Resources and Information Systems
- Web-based and Intranet-enabled medical knowledge resources
-
  - PubMed
  - Harrison's Online
  - PDR.net (the online version of the Physicians' Desk Reference)
  - Clinical Pharmacology 2000
  - Joint Commission on Accreditation of Healthcare Organizations (JCAHO) Comprehensive Accreditation Manual for Hospitals 2000
  - 1999 Hospital Statistics from the Greater New York Hospital Association
  - STATRef
  - InfoTrak
  - Dialog@CARL
  - Centers for Disease Control and Prevention, including Travel Advisory
  - AIDS Treatment Information Service
  - New York City Department of Health, including West Nile Virus updates and restaurant inspection reports
- Nutrition information
- Searchable phone book, an Access database of the global address book from Outlook
- Physician resources ('Physician Gateway')
-
  - Order sets and clinical practice guidelines
  - Patient education
  - Pharmacy newsletter
  - Health care-related web links, such as MedCalc 3000
  - Highlighted links to resources of particular importance to New York City, for example, West Nile Virus, HIV/AIDS, Lyme Disease
  - Palm Pilot downloads, such as ePocrates.com
- Training
-
  - PowerPoint presentations converted to web pages
  - Self-instructional training on conscious sedation with an online post-test
- Discussion group for the Information Systems Help Desk
- Suggestion boxes

## Results

### Intranet Traffic Report

At the time of writing, the Intranet has had 890,253 hits with an average of 3,091 hits per day. The total number of visitor sessions was 24,251 with an average of 84 per day. The total number of unique visitors was 819, of which 74 per cent (608 of 819) had visited more than once. About a third of the visitors (276) visited 10 or more times. The greatest proportion of total visitors (45%) viewed three pages.

Excluding hits to the home page, the departments receiving the most visitor sessions (defined as greater than 1,000 visitor sessions) were the 'Library' (6,130), 'Physicians Gateway' (2,539), 'Marketing' (1,321), 'Information Systems' (1,241), and 'Nutrition' (1,221) (see Table 2).

**Table 2:** Departments Receiving the Most Visitor Sessions

**Department**	Visitor Sessions
Library	6130
Physicians Gateway	2539
Marketing	1321
Information Systems	1241
Nutrition	1221
Administrative Manual	715
Help Desk	692
Human Resources	579
Phone Book	475
Training	462
Quality Assurance	450
Success Agenda	426

The average number of visitors per day was 105 on weekdays and 59 on Saturday and Sunday combined. The most active day of the week was Wednesday and the least Saturday. The most active hour of the day was from 2 PM to 3 PM and the least from 5 AM to 6 AM.

## Discussion

The Intranet has succeeded in becoming a tool used on a daily basis throughout the enterprise. It serves both business critical and patient care functions. The Intranet garnered more than 800,000 hits in its first phase. More than 500 visitors have visited more than once. Content at the end of this first phase includes a gamut of resources from an administrative manual to online training.

This mirrors the success of Intranets elsewhere in healthcare. Intranets have been used to support clinical practice guidelines [3], radiology test results [4], disease management [5], paging services [6], and a link between emergency departments [7].

The project costs in resources, software, and hardware were modest in comparison with other similar Intranet projects [4]. An existing network, server, and Microsoft software license cut Intranet project costs. The 0.7 FTE resources, less than $70,000 total for the first year, were in-sourced. In contrast, one price advertised on the web for Intranet start-up design and development is $7 per user per month . At this price, an Intranet distributed to the 5,000 users in phase one of our project, could have cost $420,000 [8].

One limitation of our analysis was the inability to determine use by different segments of the audience. Intranet was designed to permit access by all staff and associates ('Anonymous browsing allowed' in Internet Information Server 4.0). Individual logon was not necessary for access. We were therefore unable to stratify use in terms of physicians, nurses, administrators, and others.

### The Challenges and Solutions

"Build it, and they will come." It may have worked for Kevin Costner in the movie "Field of Dreams", but chances are this philosophy alone won't work for your company's Intranet. - P.G. Daly [9].

As with any innovation, at the outset, the Intranet was not adopted immediately [10]. Early adopters provided content to the Intranet and promoted its early use by physicians. The medical library director asked that web-based and web-enabled resources be put online. The clinical chair of medicine requested that the physician order sets and clinical practice guidelines be made available online.

Content spurred use by other staff groups. The nursing coordinator for patient education worked to create material for the most common conditions and illnesses. The Nutrition department submitted training presentations.

The project team webmaster and leader actively sought content and marketed the Intranet in one-on-one meetings with department and corporate leadership. As influential early adopters made the Intranet their own, more stakeholders and departments showed interest in the Intranet.

Though adoption may have been the principal barrier, solutions for other challenges were no less critical to the Intranet's performance and survival. The Intranet project team faced a number of challenges.

- The imminent merger presented a barrier to some who were unclear what their own role or that of their department might be in the new organization. They were hesitant to commit time and resources to a venture they might not see complete. For others, creating an Intranet site for their department fostered collaboration between the regions and served as a means to advertise the products and services they offer to the enterprise.
- Timely receipt of content was particularly based on promoting existing or building new interpersonal relationships. Lack of familiarity with Intranet functionality and questions about how departmental content would be managed were two often voiced concerns. The webmaster developed a PowerPoint presentation that introduced Intranet technology and described its potential to enable the business and patient care missions of the enterprise. Communication skills were as critical as programming knowledge. A policy was developed that required that all content have the approval of the relevant departmental leadership.
- Converting corporate pamphlets, leaflets, and newsletters required obtaining ASCII or word processed text from the various contracted print publishers. Marketing was encouraged to send soft copies to the Intranet webmaster whenever new print media was created. To prevent delays in publication and to reduce development turnaround time, text was accepted only in digital format. Photographs were accepted in Graphic Interchange Format (GIF) or Joint Photographic Experts Group (JPEG) format but could also be sent in hardcopy and scanned.
- Collapsible guidelines, dynamic HTML, and other features at the time were only supported by Internet Explorer versions 4 and above. Internet Explorer 5 was made the standard web browser. Web browser standardization was accomplished with Microsoft Systems Management Server.
- To enable collaborative authoring and work team sites with restricted access, security permissions for directories (access control lists) and files were managed in the Windows NT file system (NTFS).
- Marketing the Intranet meant more than announcing its existence in corporate email broadcasts and newsletters. The Intranet team met with departmental leadership in locations and times convenient to them. Meeting preparation required a checklist of several items. We confirmed network connectivity and installation of Internet Explorer 5 on suitable workstations before each meeting. The Intranet was made the default home page on these workstations and a readily apparent shortcut placed on each desktop screen. If a user did not have an account on the new enterprise-wide domain, a new account was made or an old account was moved.
- Success inherently generates problems of its own. As more departments adopt the Intranet, more content is created that must be re-formatted to web-browser friendly layout and incorporated into the web architecture. The increase in content may be temporary and related simply to the recent merger.

If the content submission and turnover continue to grow, several options will be considered. We are currently experimenting with a method that permits Marketing to publish content directly to the Intranet without the need of an intermediary, for example, form posting to an HTML file. Alternatively, content submission and development could be limited to once or twice a month rather than weekly. Finally, more resources may be needed in Intranet design and development.

### Next Steps

With successful completion of the first phase of the Intranet project, the second phase will have three goals:

1. Re-engineer the Intranet content and format to serve the new enterprise in all regions. New sites will include the physicians-hospital organization, the nursing department, and JCAHO readiness.
2. Grow Intranet application development, for exempt, enterprise-wide job postings, podiatry procedure log, uniform formulary database.
3. Extend the web browser standard and validate network connectivity throughout the enterprise.

### Conclusion

Intranet technology is readily available and is, if pre-existing resources are already in place, of modest cost. Adoption was the key barrier to diffusion in our project. By demonstrating how the Intranet can serve business critical and patient care needs, we were able to empower early adopters. The Intranet has succeeded in reaching the early majority and has evolved from a cutting edge technology to an everyday tool.

## Appendix 1

Downloadable Screenshots of the Intranet
