# Supplementary material for: Building and Growing a Hospital Intranet: A Case Study
Source: J Med Internet Res. 2001 Mar 17;3(1):e10. doi: 10.2196/jmir.3.1.e10 (PMC1761884; doi:10.2196/jmir.3.1.e10)

## Slide 1
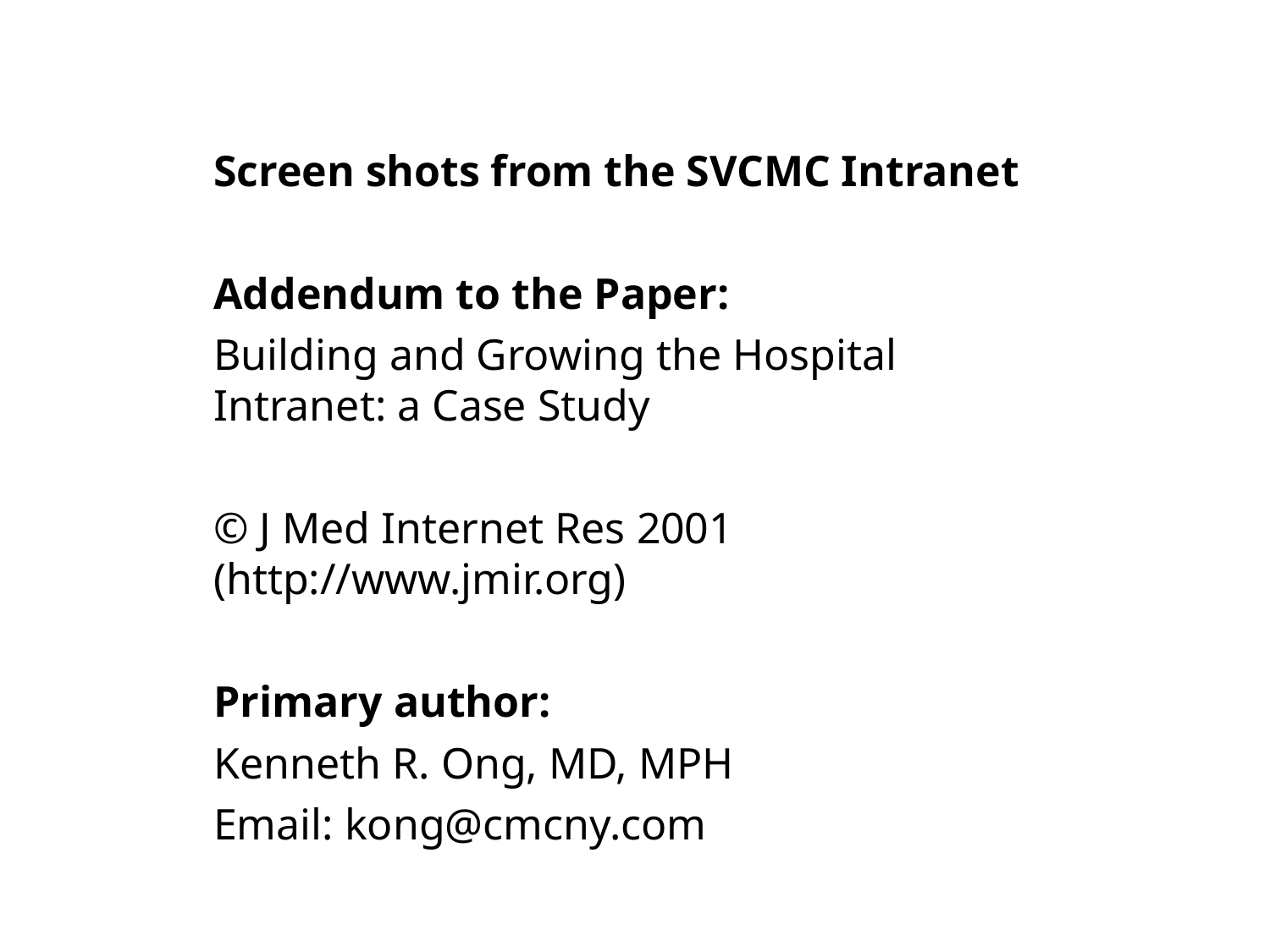

# Screen shots from the SVCMC Intranet
Addendum to the Paper:
Building and Growing the Hospital Intranet: a Case Study
© J Med Internet Res 2001 (http://www.jmir.org)
Primary author:
Kenneth R. Ong, MD, MPH
Email: kong@cmcny.com

## Slide 2
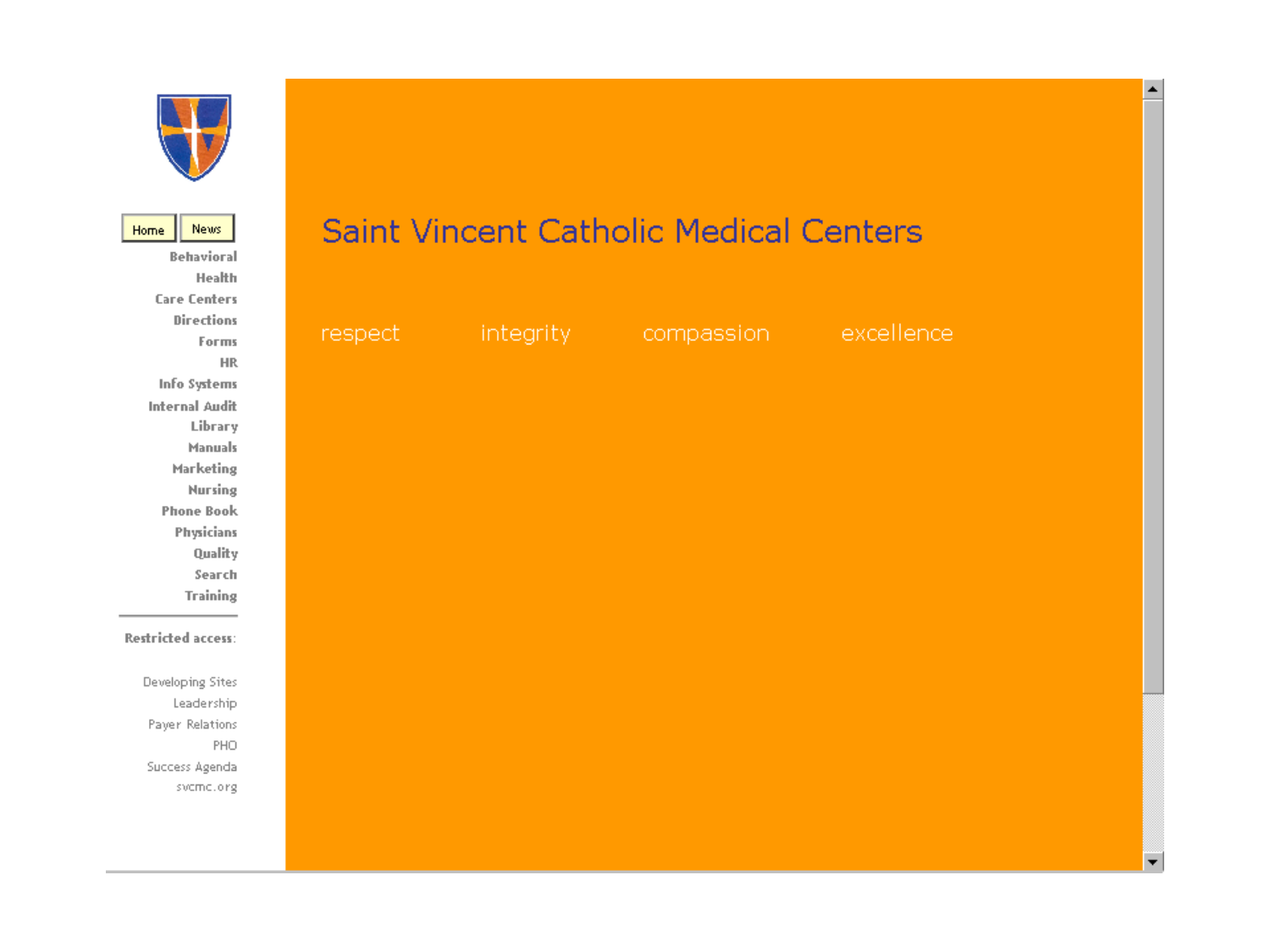

## Slide 3
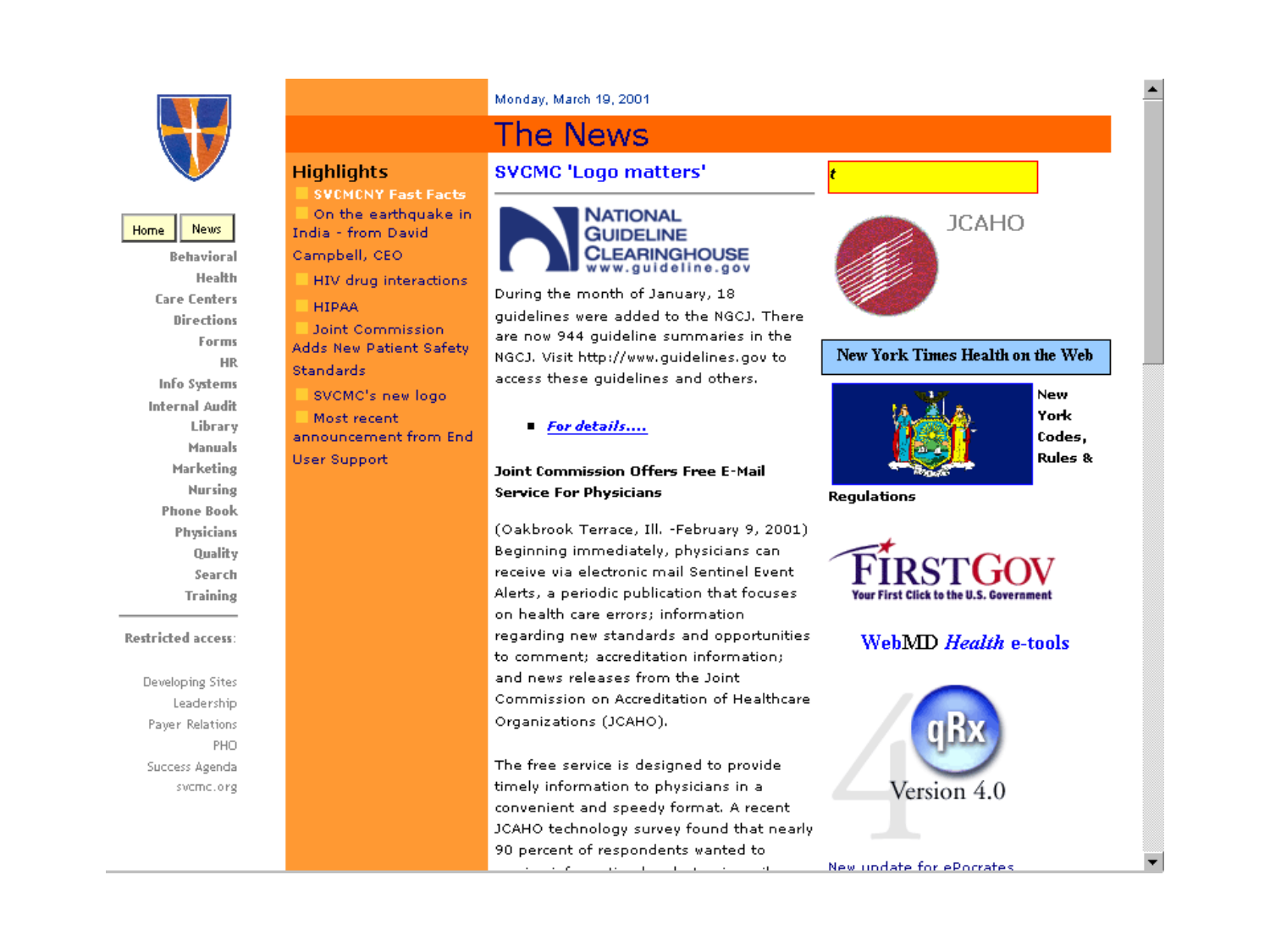

## Slide 4
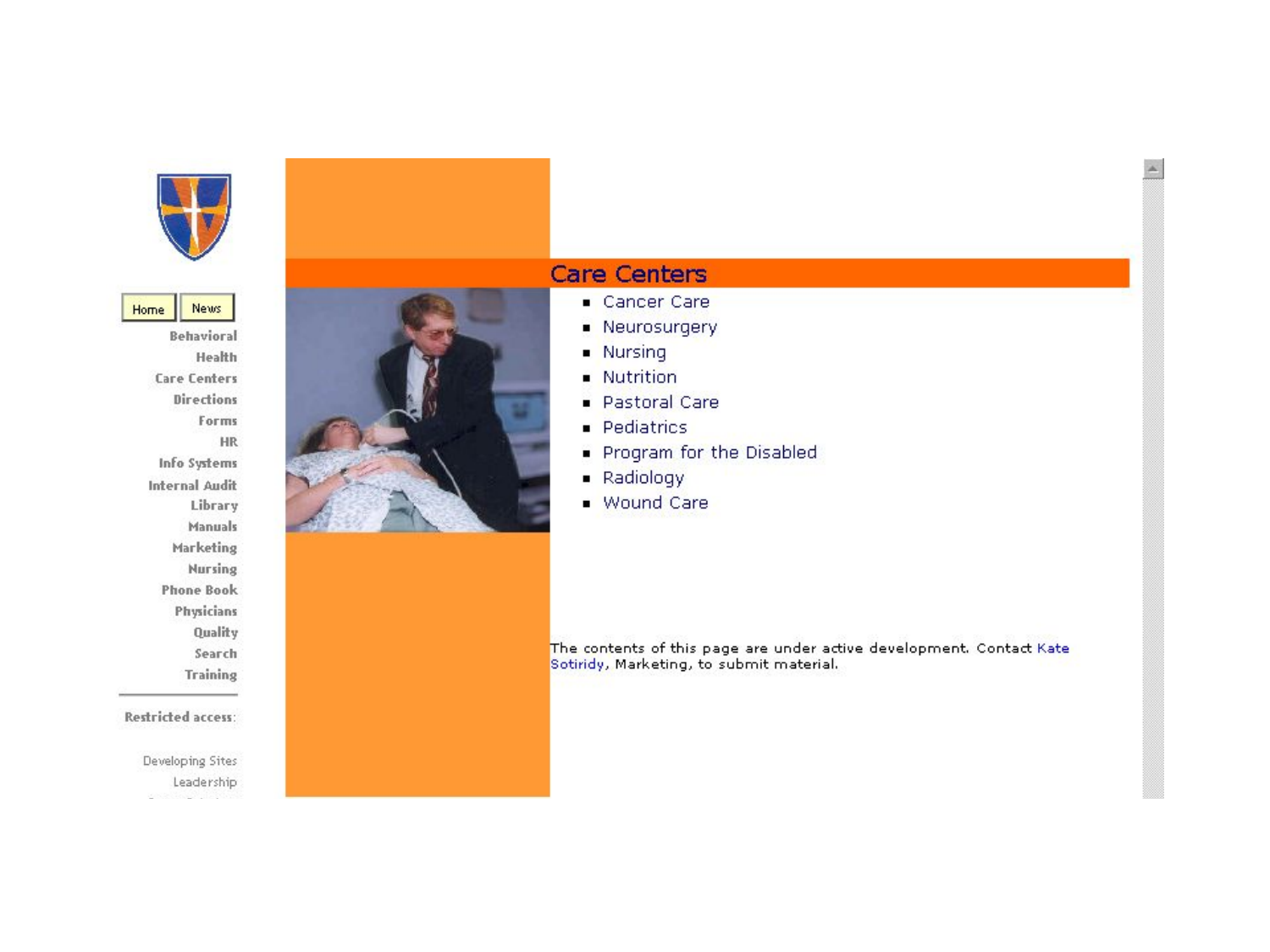

## Slide 5
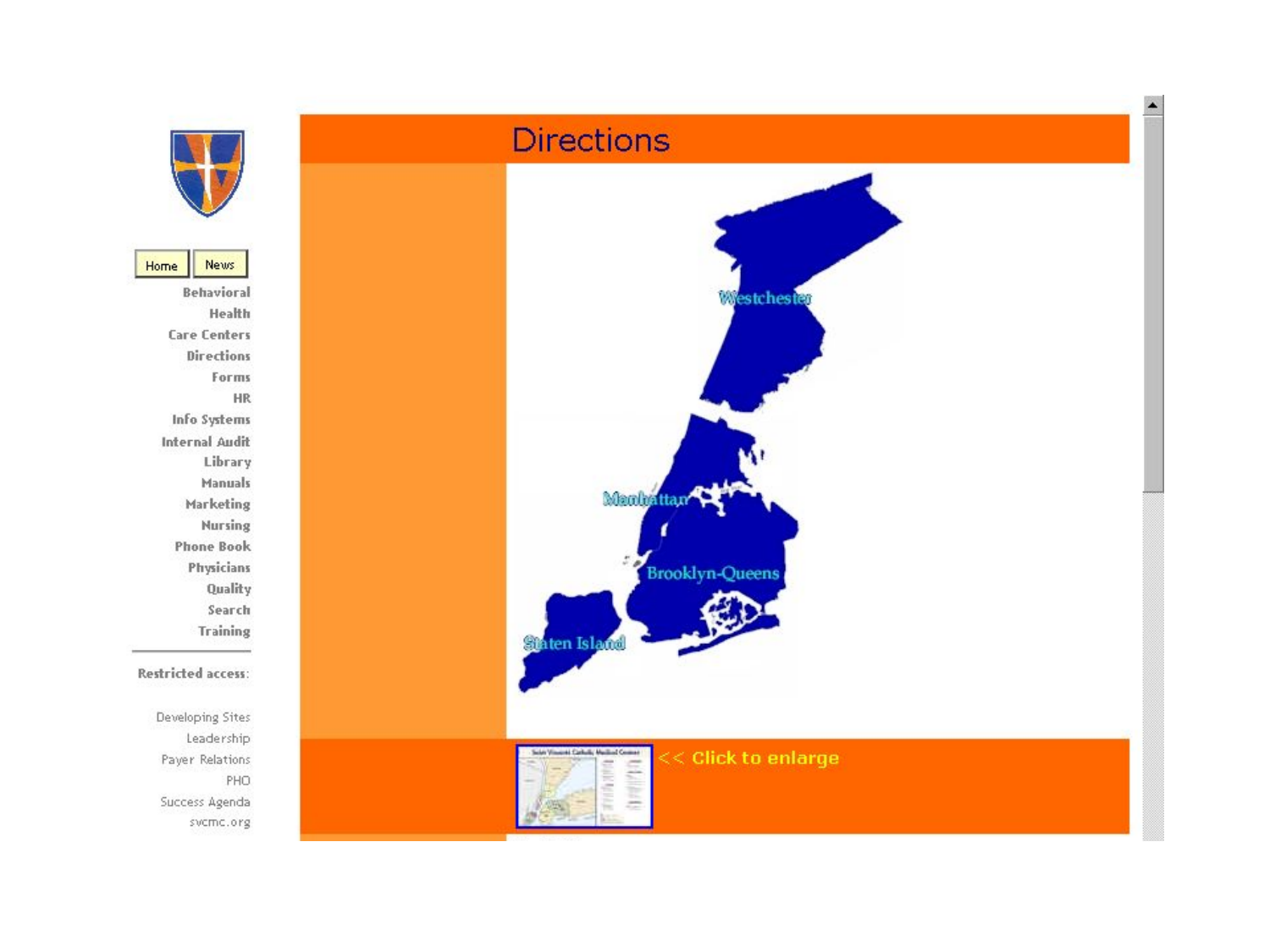

## Slide 6
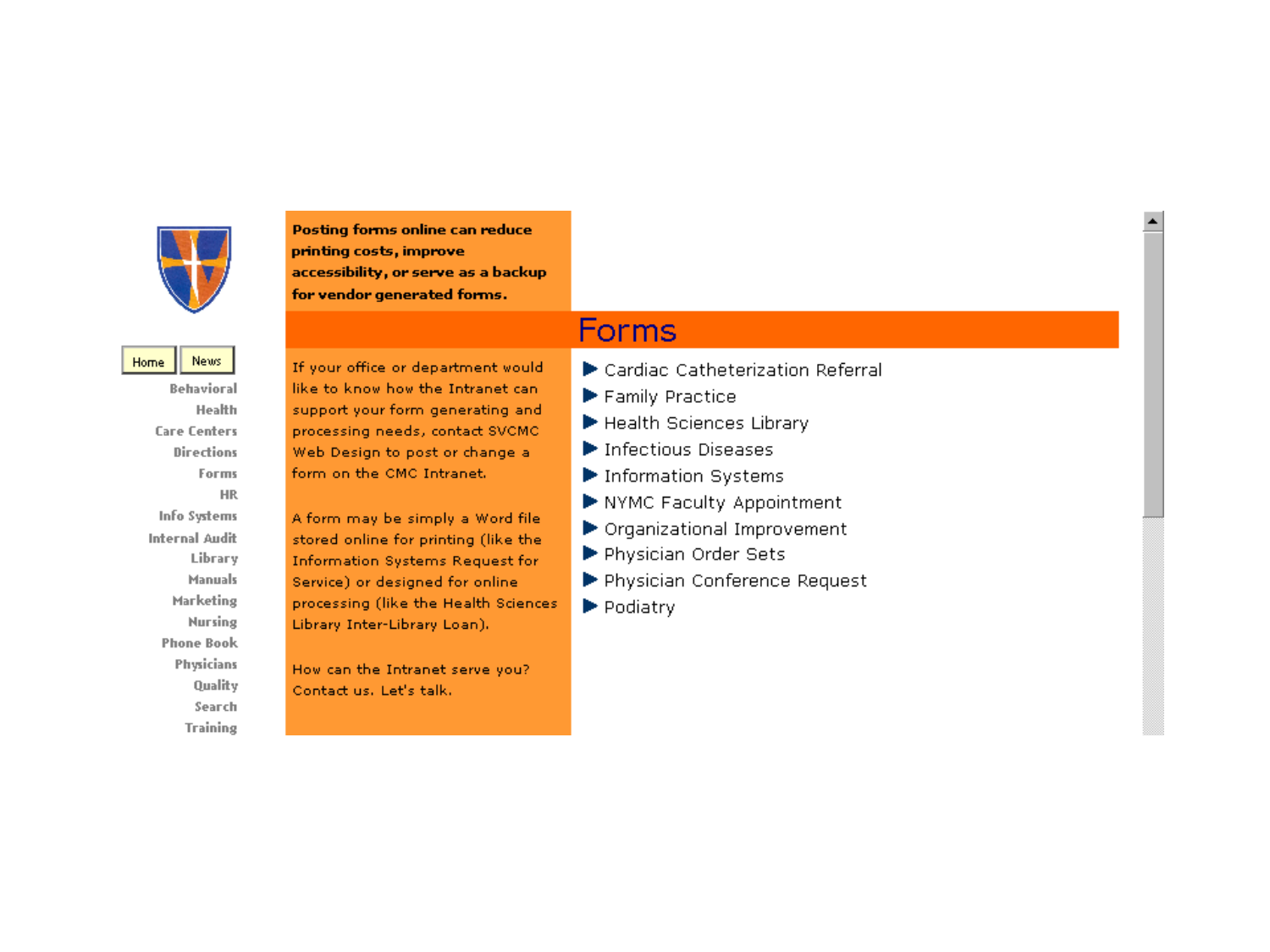

## Slide 7
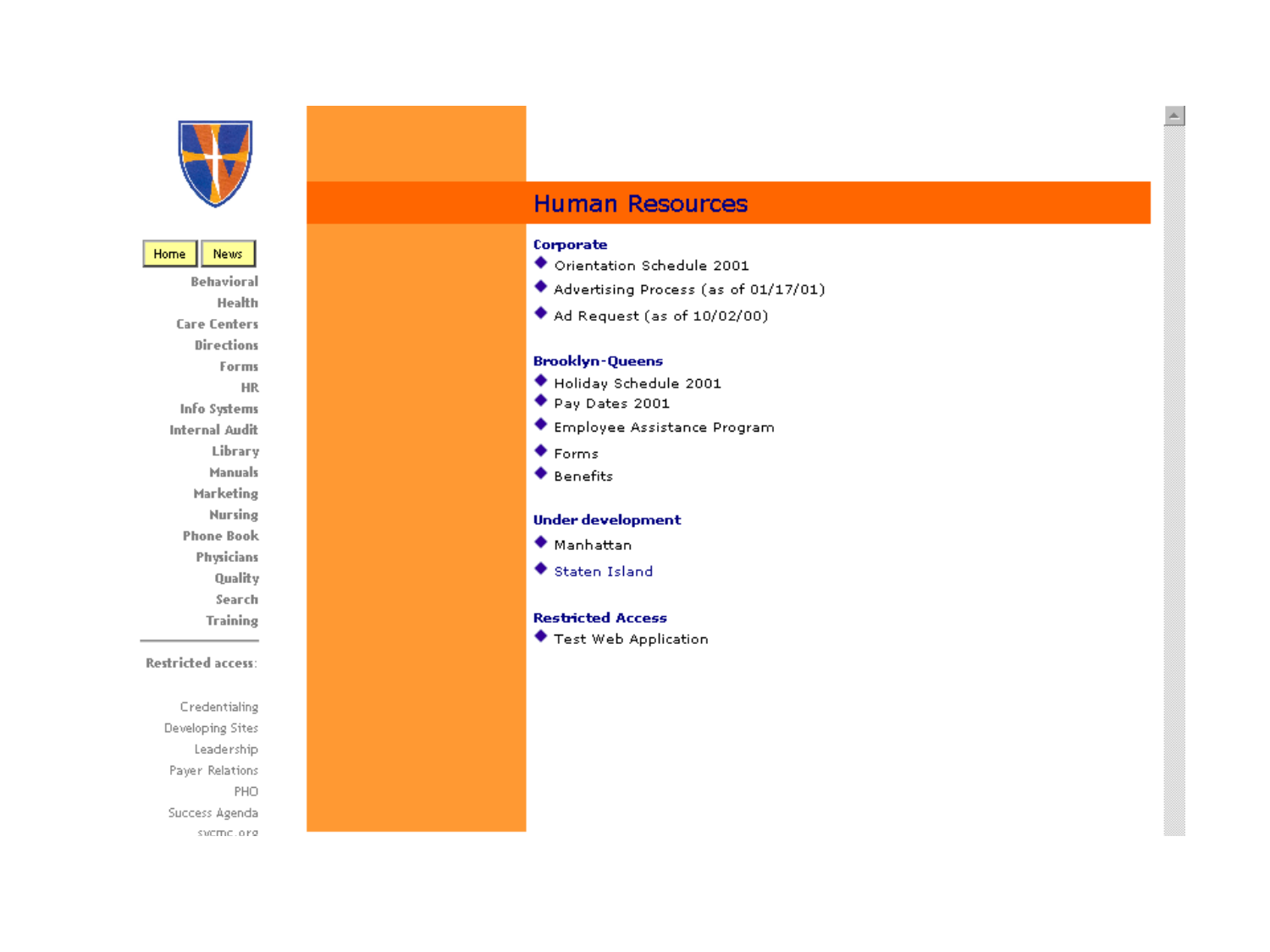

## Slide 8
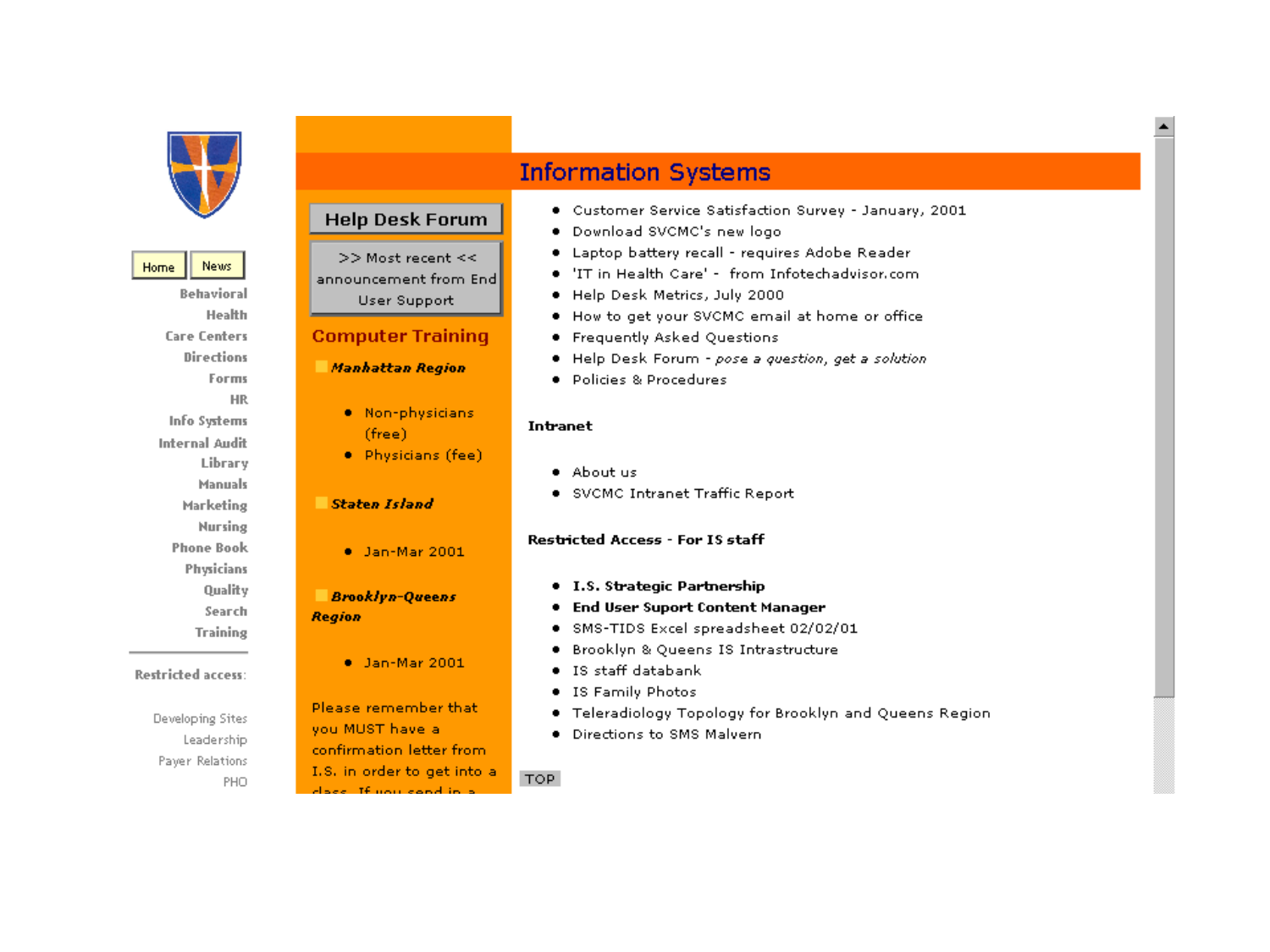

## Slide 9
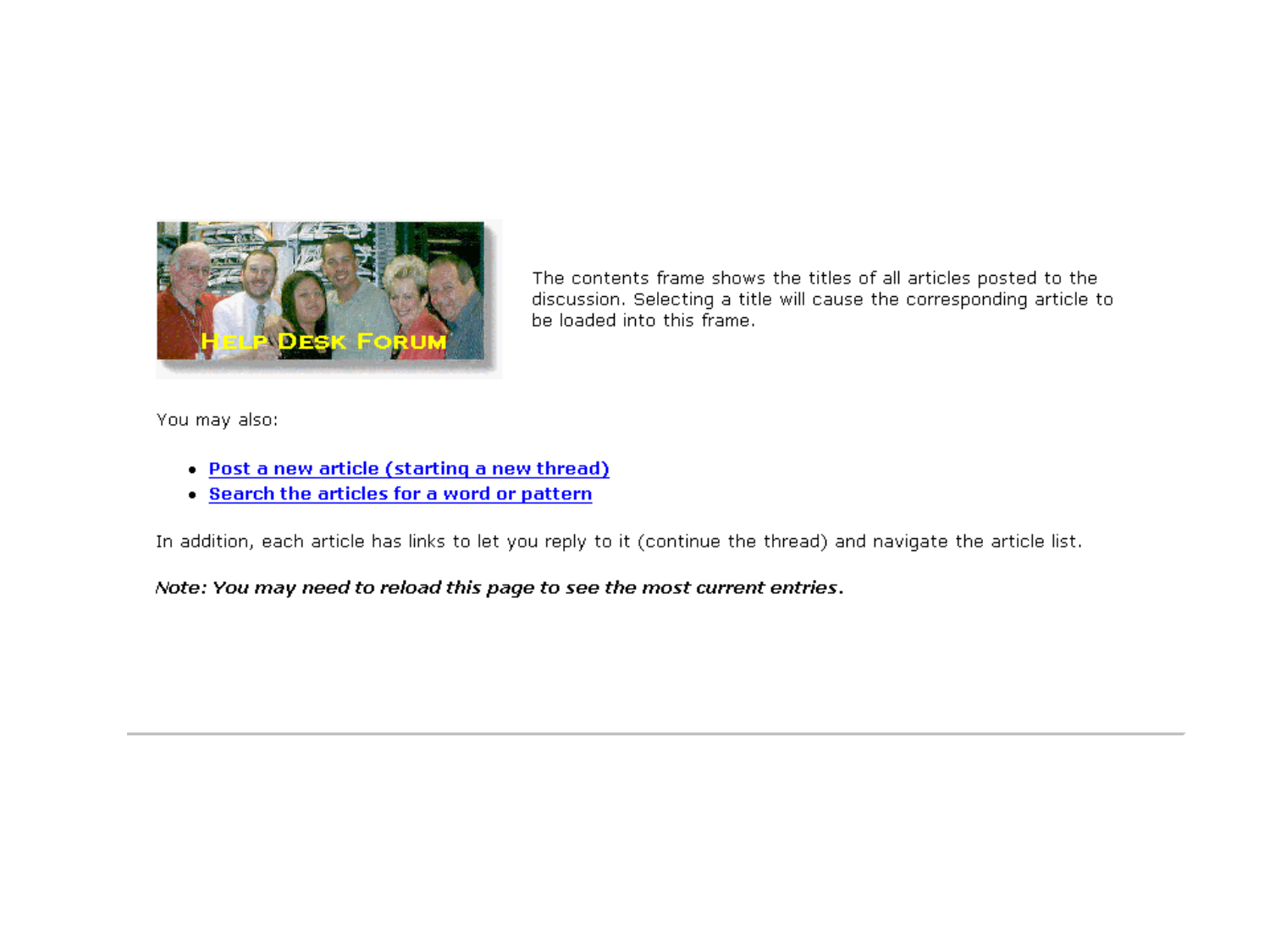

## Slide 10
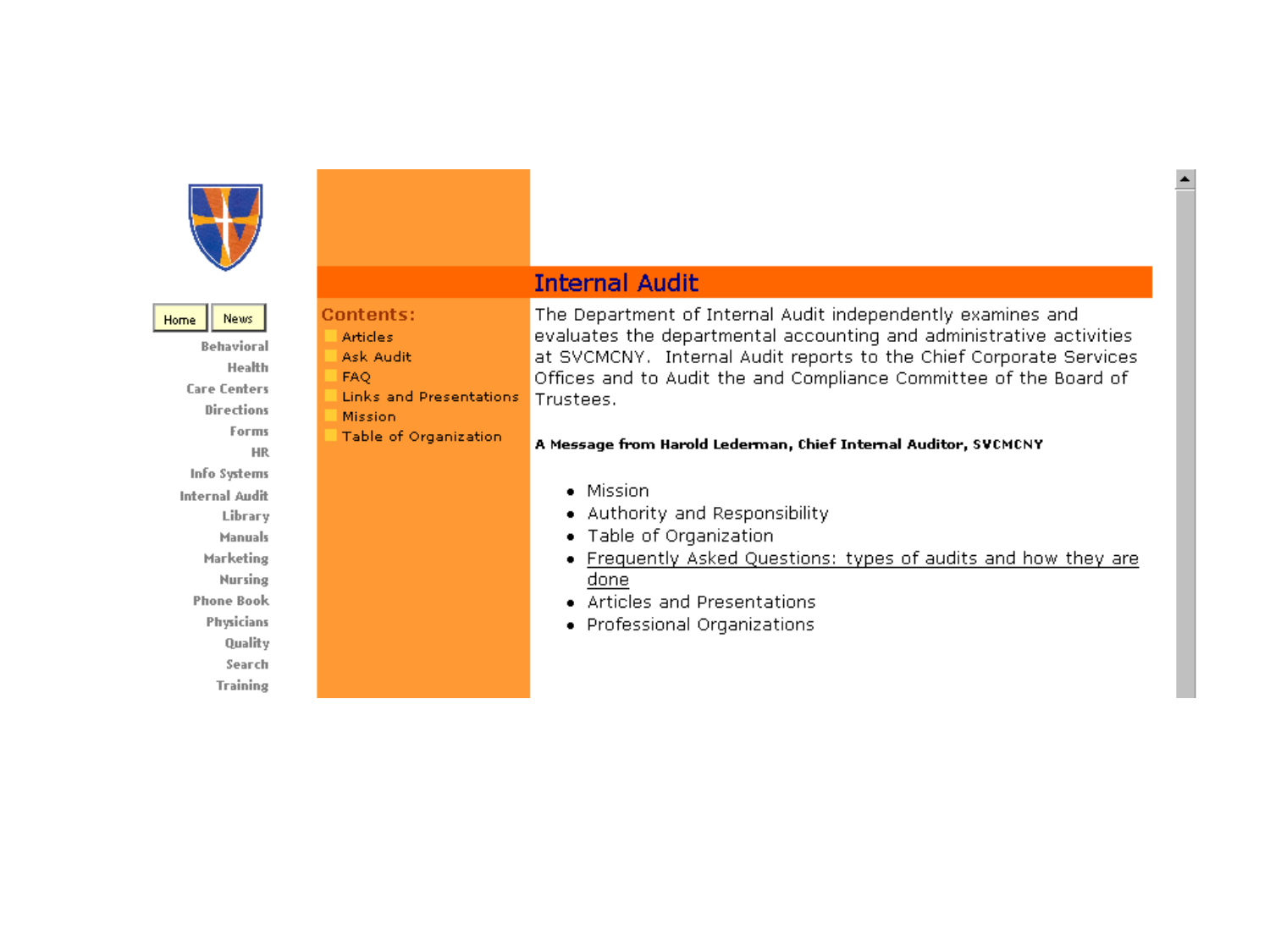

## Slide 11
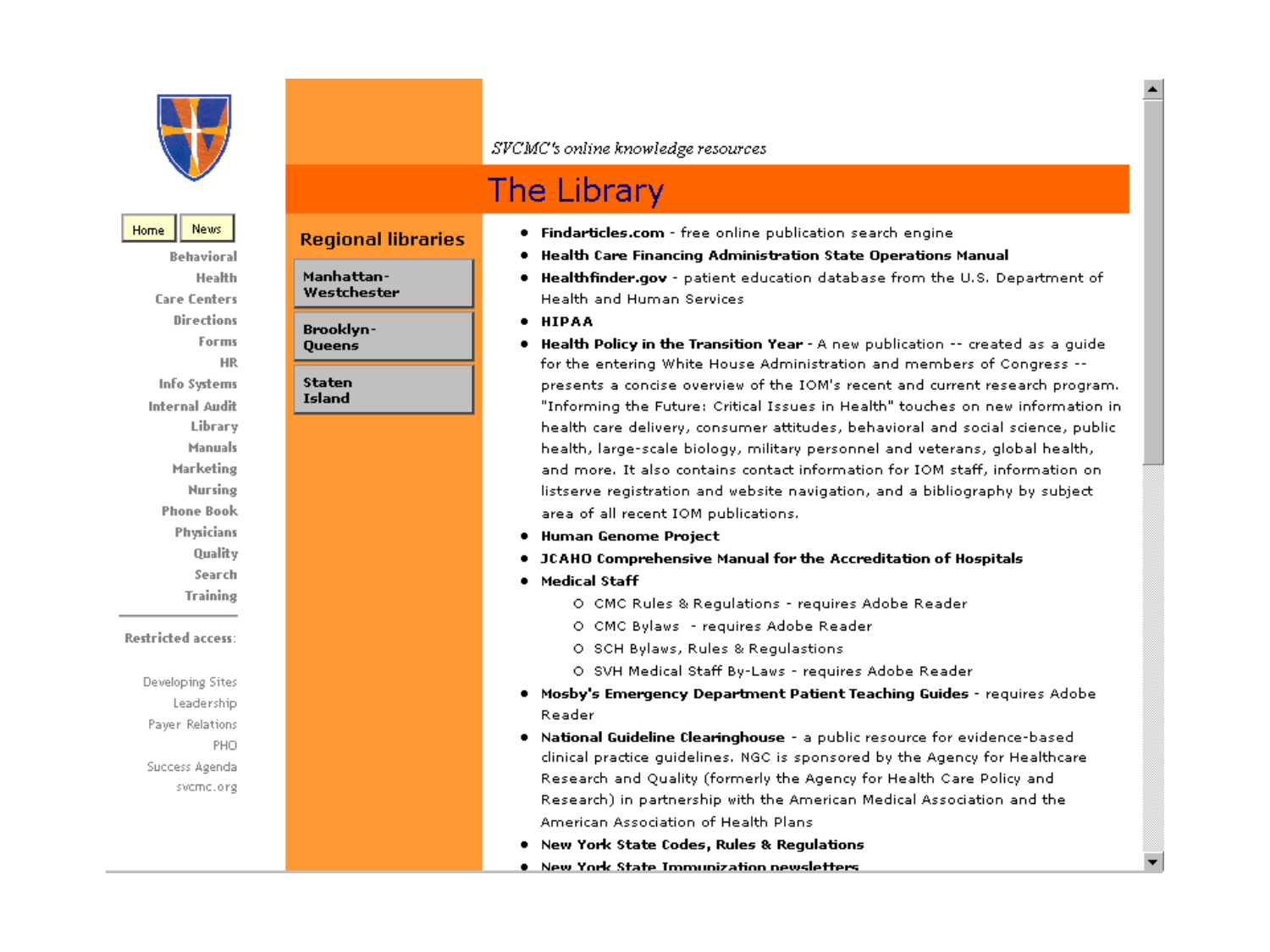

## Slide 12
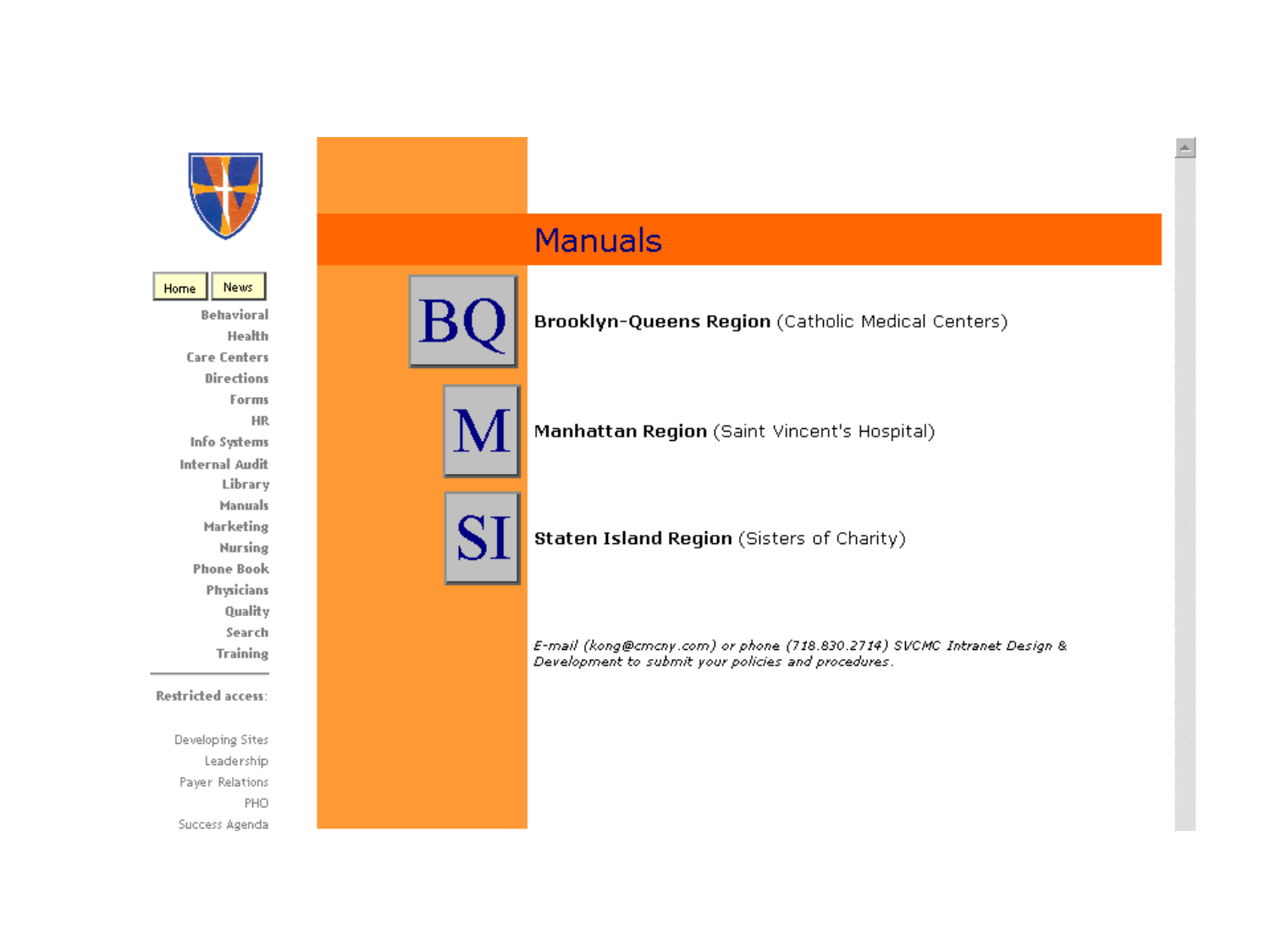

## Slide 13
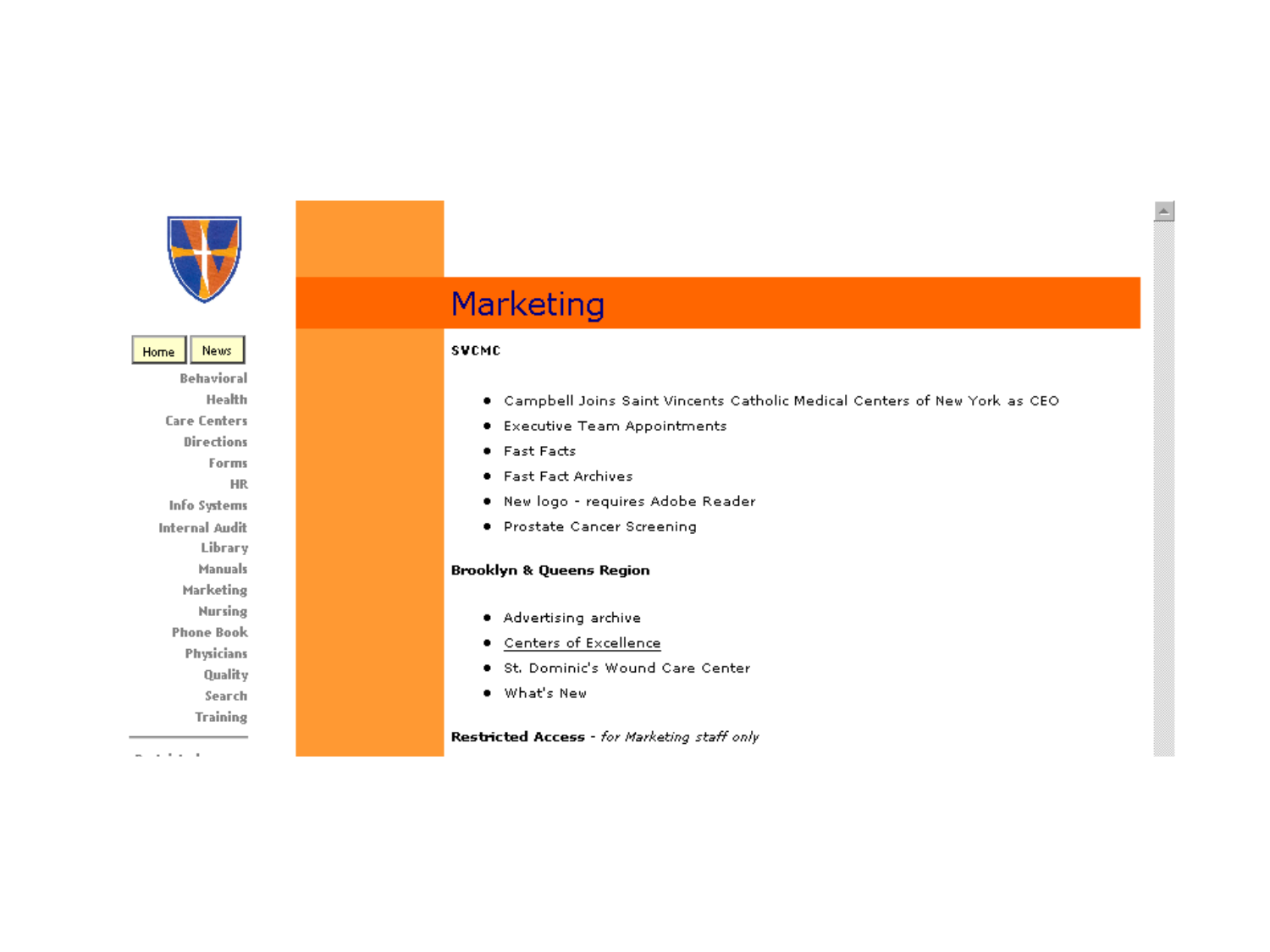

## Slide 14
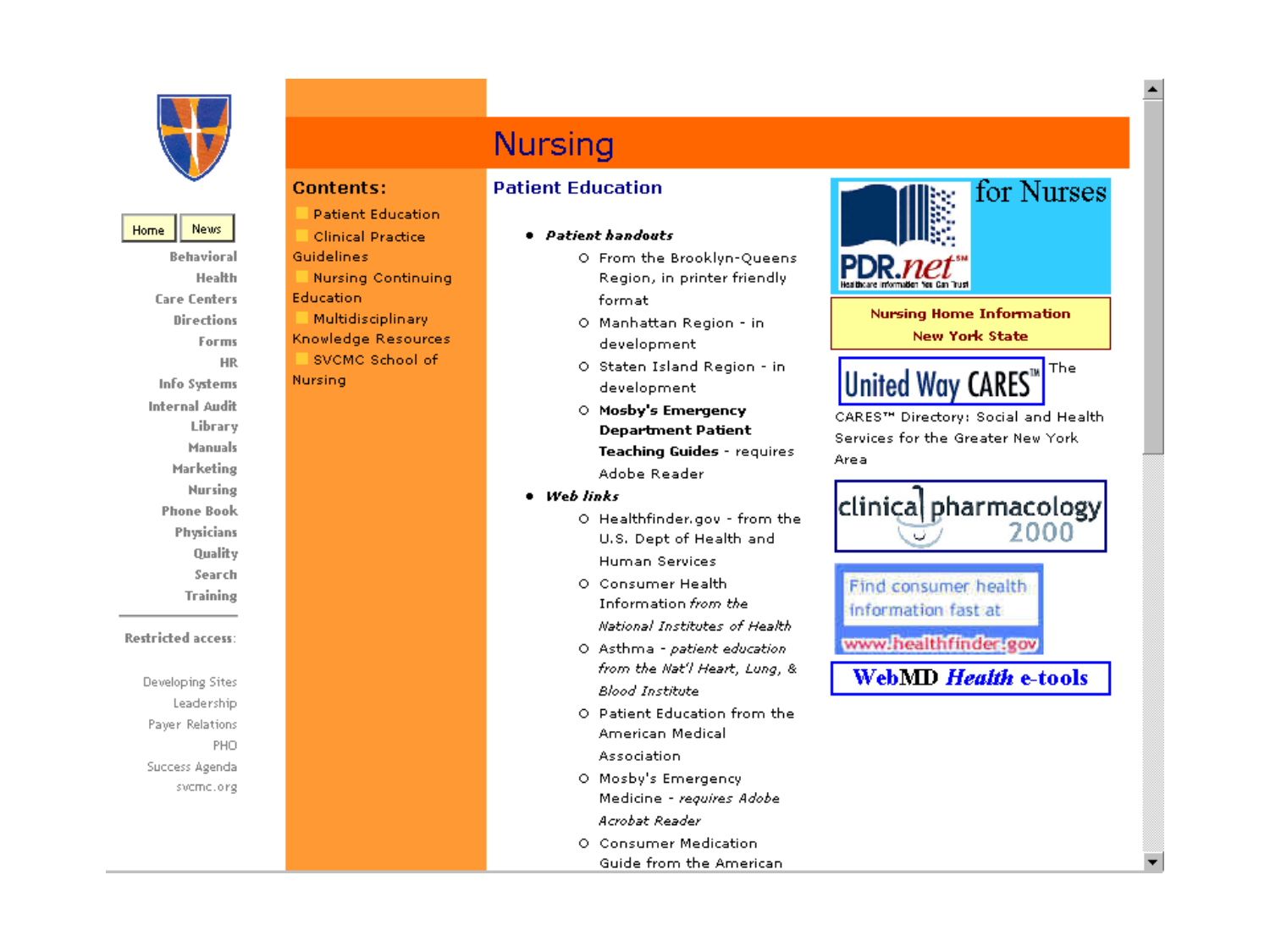

## Slide 15
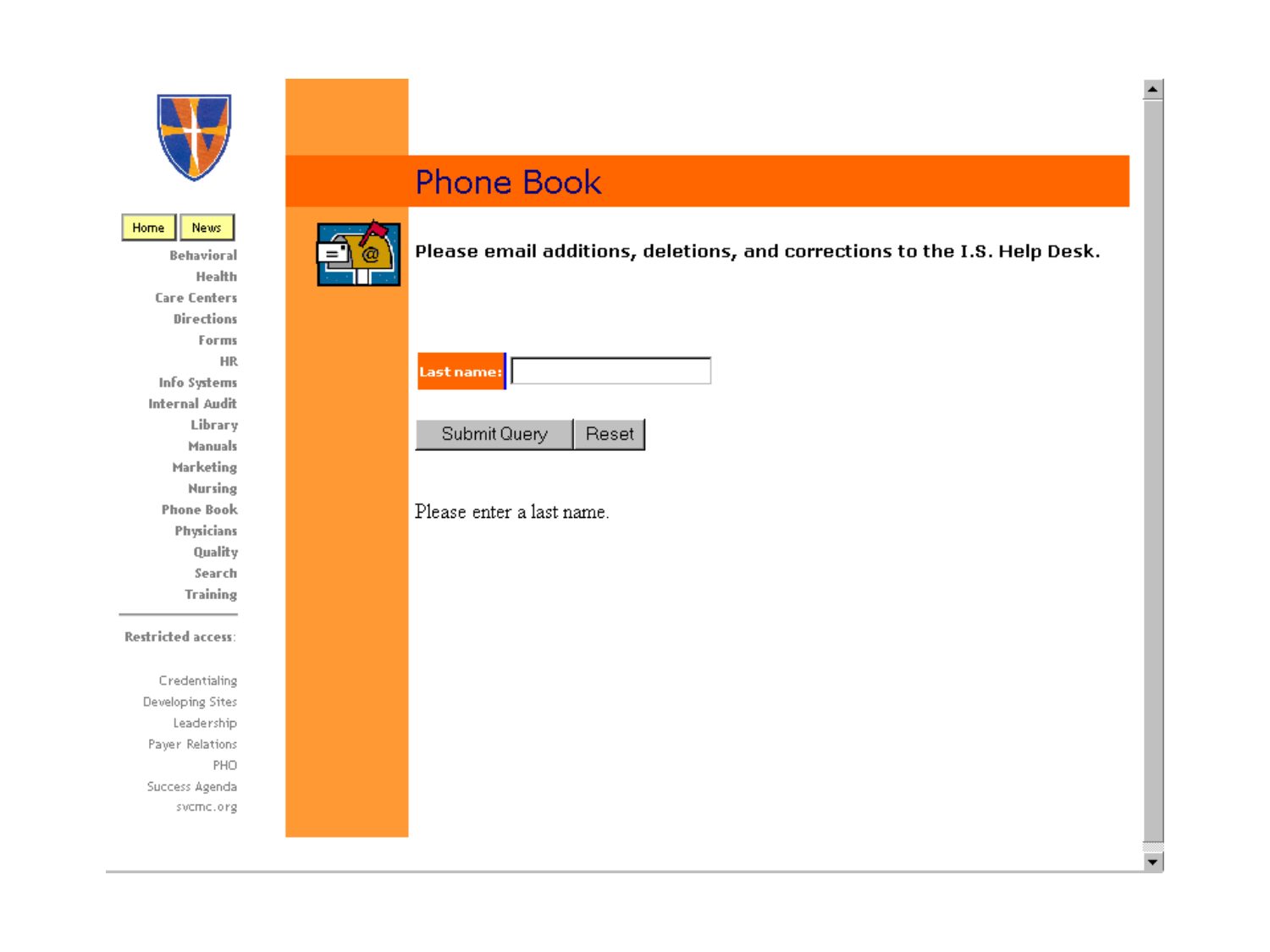

## Slide 16
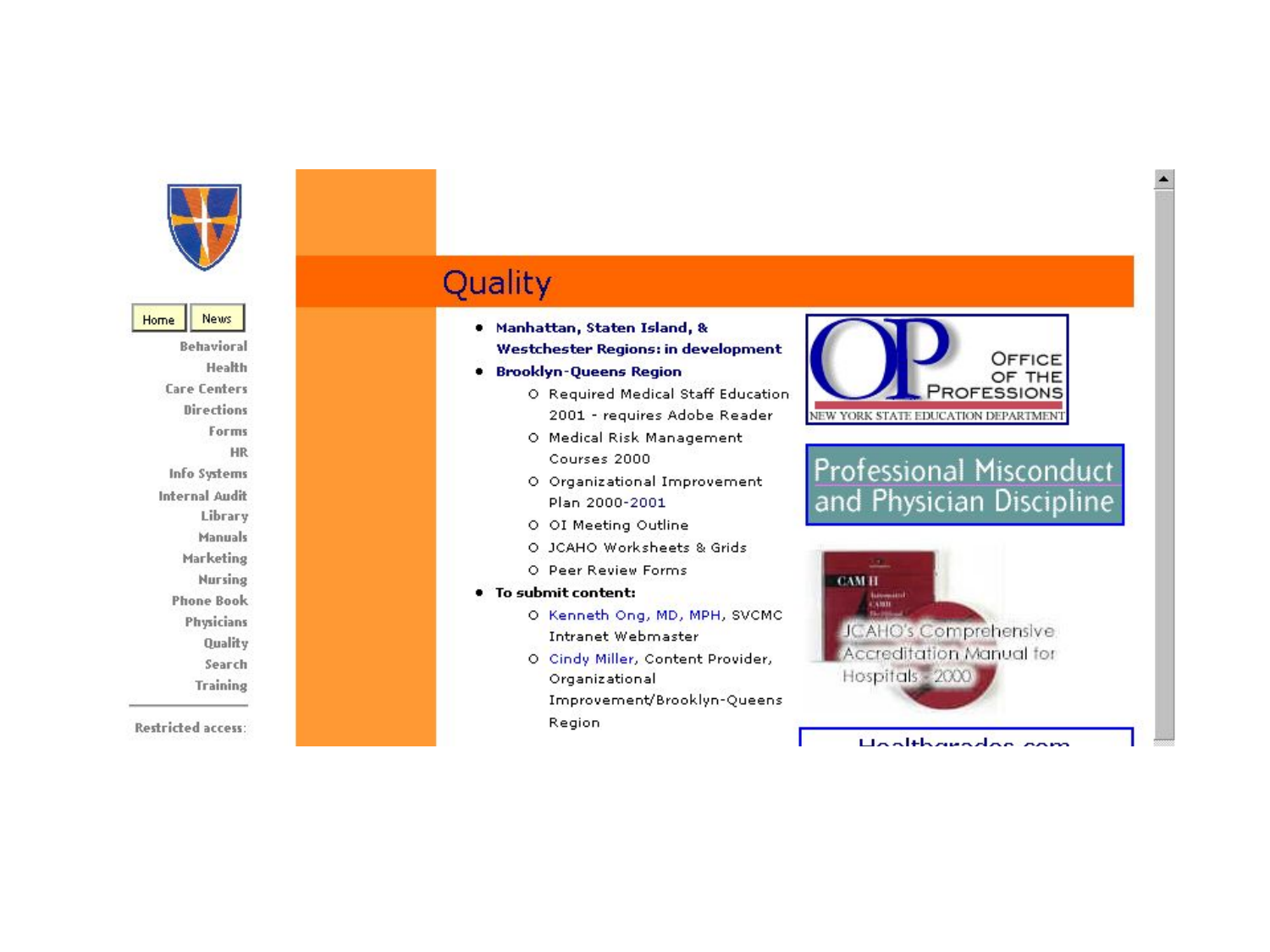

## Slide 17
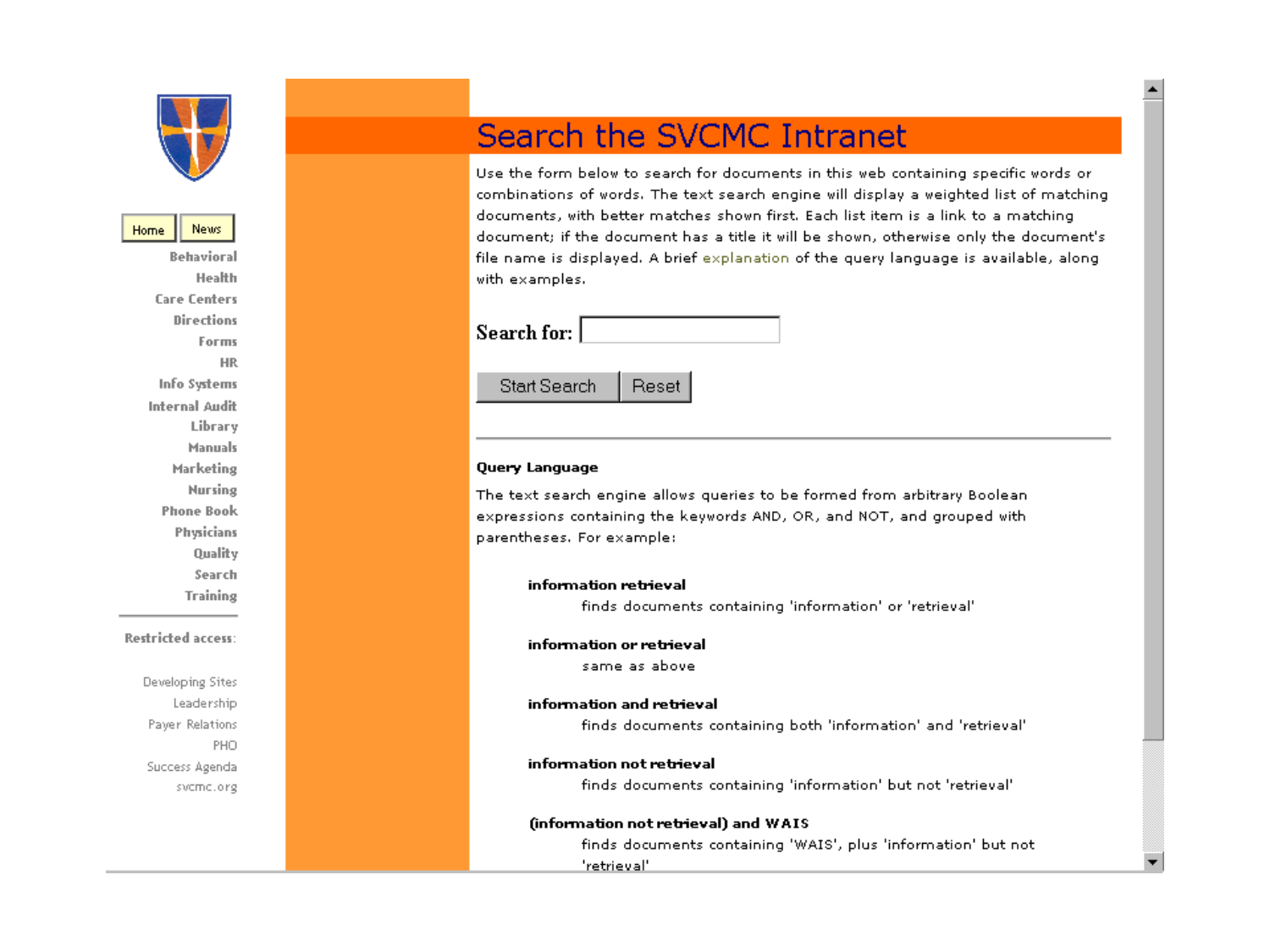

## Slide 18
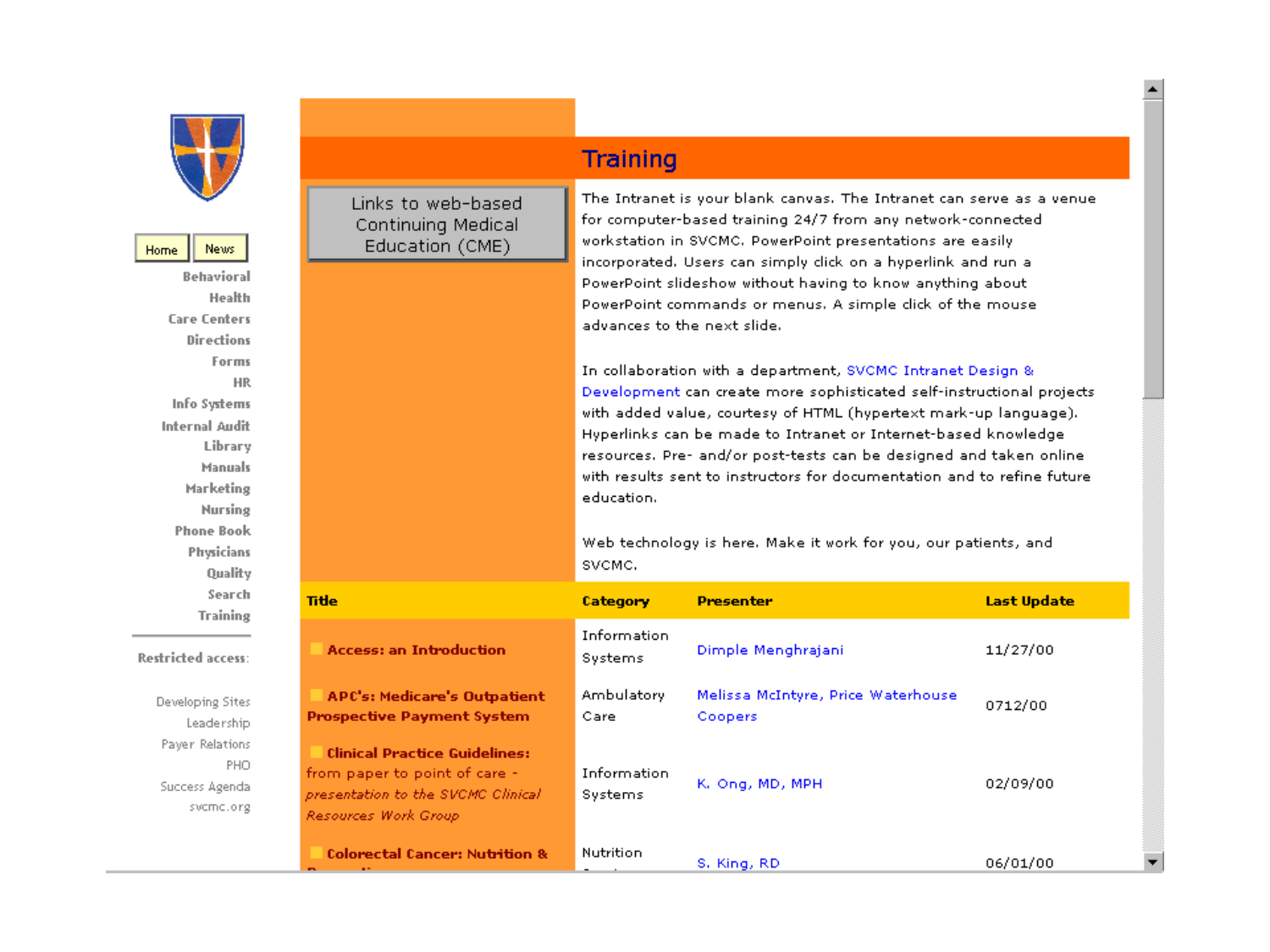

## Slide 19
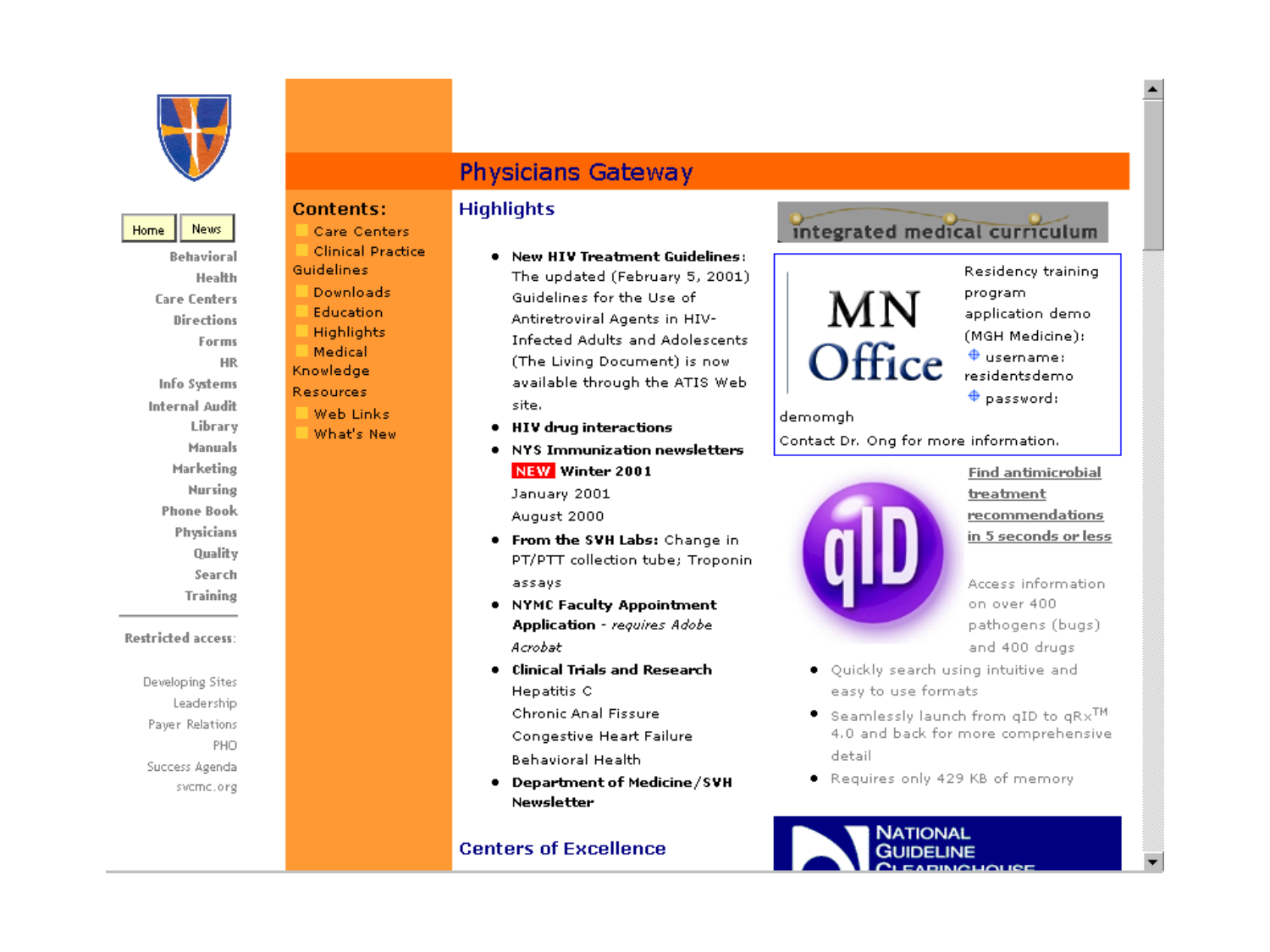

## Slide 20
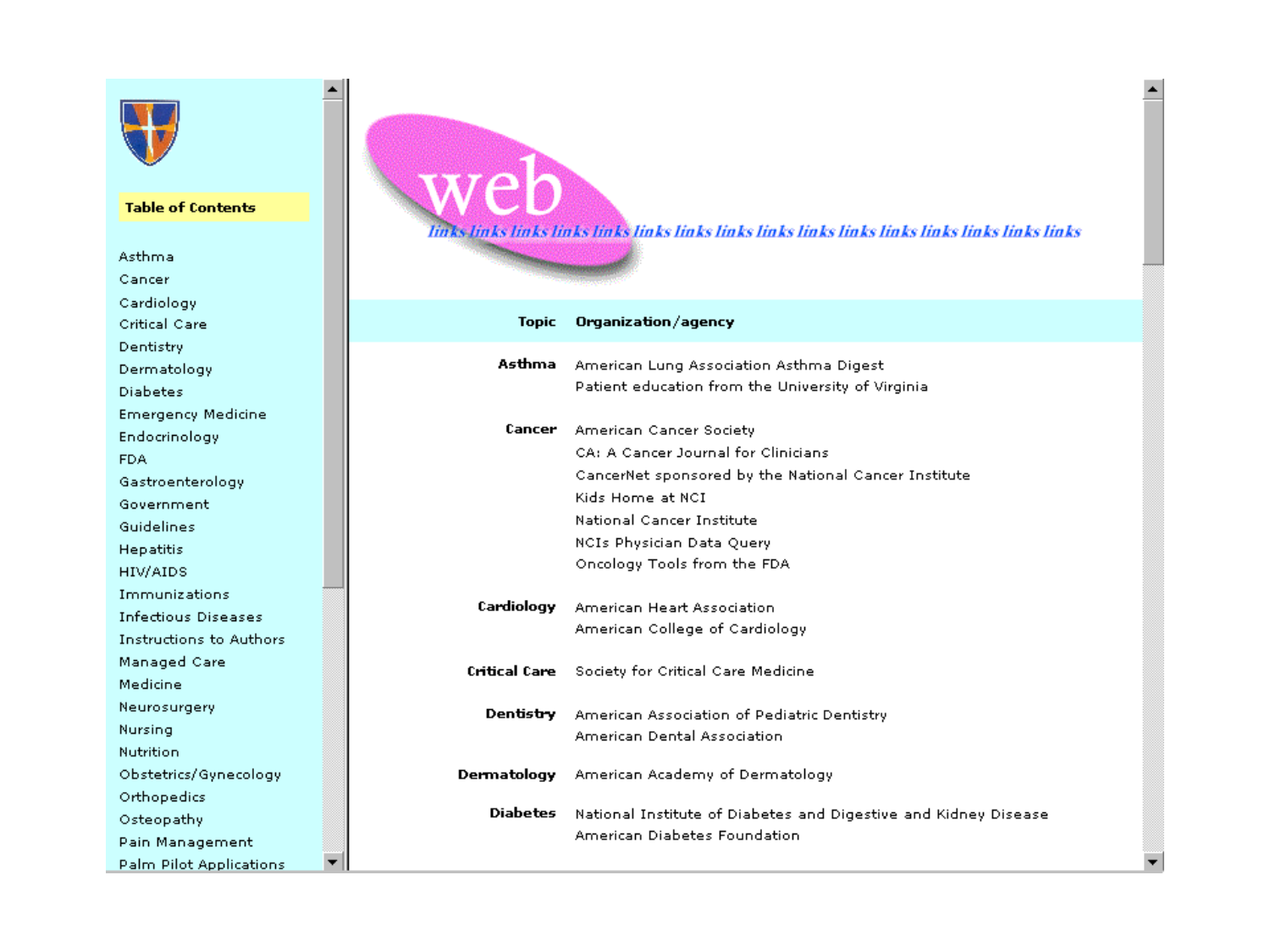

## Slide 21
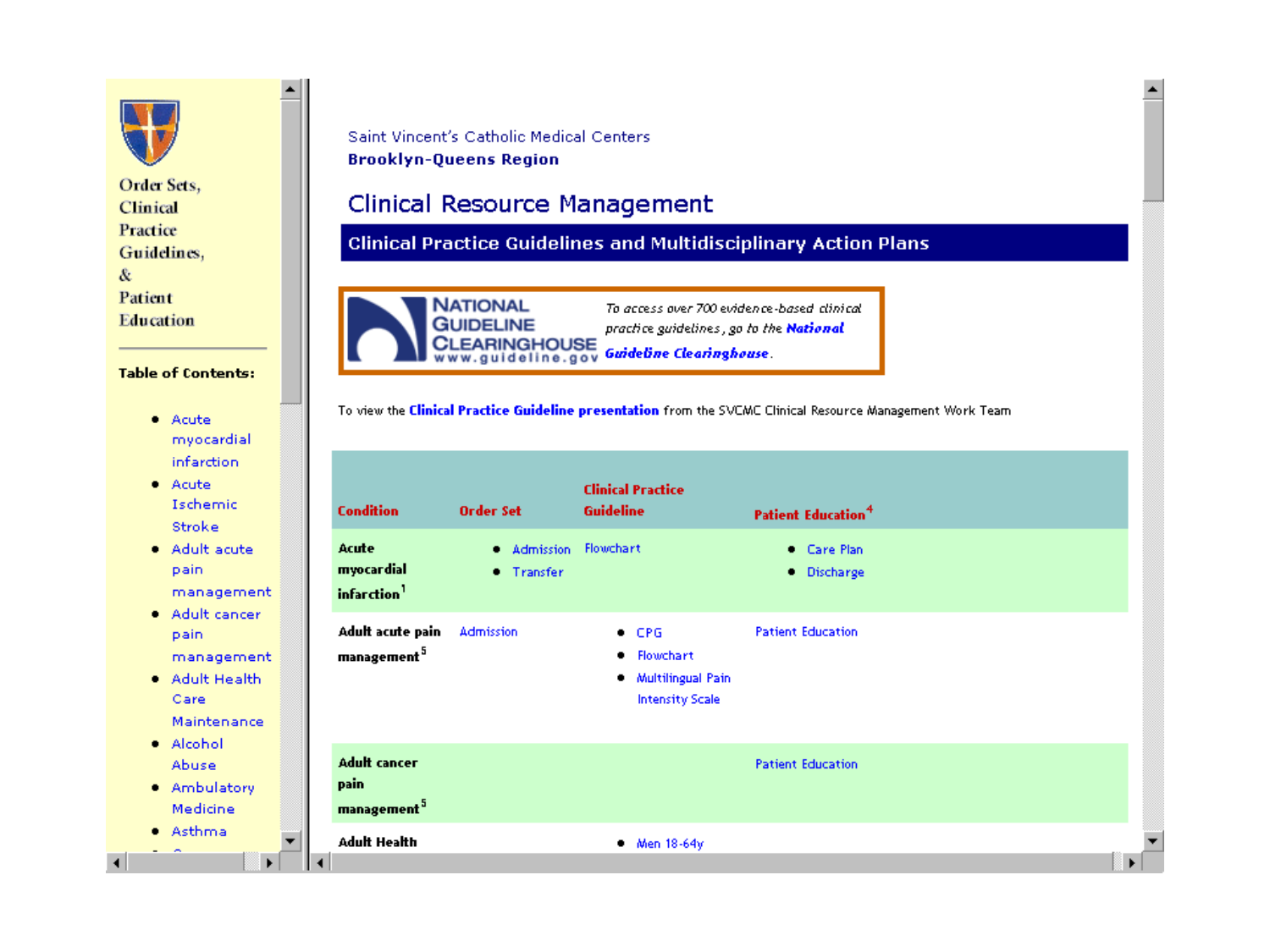

## Slide 22
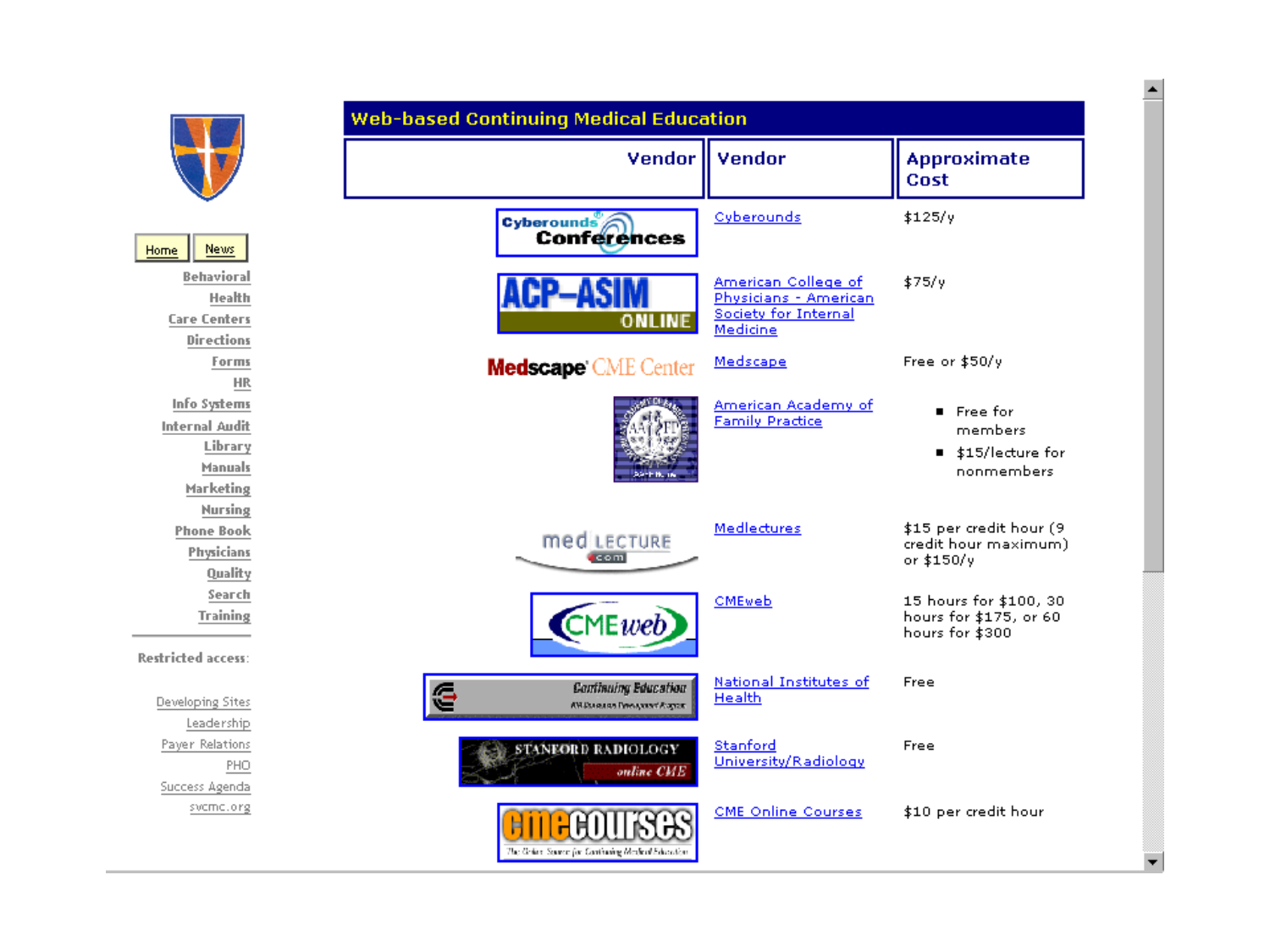

Supplement: Supplementary file 1 [file jmir_v3i1e10_app1.ppt]
